# Applications of nutritional functional units in commodity-level life cycle assessment (LCA) of agri-food systems

**DOI:** 10.1007/s11367-019-01679-7

**Published:** 2019-09-07

**Authors:** Graham A. McAuliffe, Taro Takahashi, Michael R. F. Lee

**Affiliations:** 1 Rothamsted Research, North Wyke, Okehampton, Devon EX20 2SB UK; 2 University of Bristol, Bristol Veterinary School, Langford, Somerset BS40 5DU UK

**Keywords:** Agriculture, Climate change, Environment, Food production, Life cycle assessment, Literature review, Nutrient density score, Nutrition

## Abstract

**Purpose:**

The nutritional quality of final products is attracting an increased level of attention within life cycle assessment (LCA) literature of agri-food systems. The majority of these studies, however, are based on comparisons at the dietary level and, therefore, are unable to offer immediate implications for farmers as to how best to produce food. This article evaluates recent literature examining the nutrition-environment nexus at the commodity level, with the aim to identify potential pathways towards sustainability analysis that can inform both consumers and producers.

**Methods:**

A systematic search of literature was carried out to produce a shortlist of studies, and strict exclusion criteria were applied to them afterwards to eliminate irrelevant material. The studies thus selected were classified into one of three tiers based on the level of complexity with regard to their functional units: (1) based on single nutrients, (2) based on composite indicators derived from multiple nutrients and (3) based on commodity-level analysis in a dietary context.

**Results and discussion:**

Sixteen papers were identified for inclusion in the review. All of them accounted for climate change either directly or indirectly, whilst only five addressed different impact categories at the same time. Nine studies estimated environmental impacts under functional units associated with nutrient density scores, and the others utilised alternative approaches to account for nutritional value such as linear programming and end-point modelling combined with epidemiological data. A recently developed method to calculate the marginal contribution of a commodity to the overall nutritional value of a specific diet was considered to be a successful first step in bridging the aforementioned knowledge gap.

**Conclusions:**

The LCA community should continue the ongoing effort to link farm management decisions to diet-level environmental impacts through an enhanced focus on human nutrition across the entire value chain. Future research comparing environmental performances of multiple food groups or multiple production systems should acknowledge differences in nutritional composition and bioavailability between the final products and, ideally, the effects of these nutrients on overall dietary quality.

## Introduction

Life cycle assessment (LCA) is one of the most common and comprehensive tools for comparing environmental burdens arising from the agri-food sector (de Vries and de Boer 2010; de Vries et al. 2015; McAuliffe et al. 2016; Roy et al. 2009). However, recent literature has identified a fundamental issue associated with the selection of functional units in many of these comparisons. Food-based LCA studies typically utilise functional units based on mass or volume of a given product rather than the true function of the commodity which is to provide nutrition (Van Kernebeek et al. 2014). Heller and Keoleian (2003) were pioneers in acknowledging that food consumption patterns should be incorporated into the LCA framework when they recognised sustainability-limiting factors such as rapid conversion of prime farmland (economic), excessive depletion of topsoil (environmental) and illegal farm operatives (social) in the US food system. More recently, Heller et al. (2013) reviewed work carried out over 10 years since their 2003 publication and proposed key areas which require further investigation. The authors noted that considering food quality, here defined as nutrient contents and composition, is critical to improve understanding of the food-environment nexus. Whilst a growing body of research has been addressing this methodological roadblock (Nemecek et al. 2016; Schau and Fet 2008), a consensus on how best to navigate it has not been met.

At the simplest level, the LCA community has tackled the issue of nutritional composition from two directions: diet and product. Of the two approaches, dietary LCA studies have become widespread over the last decade, and several reviews have subsequently assessed their prominence. Van Kernebeek et al. (2014), for instance, explored assessments of diets from 12 peer-reviewed papers which compared varying degrees of meat consumption with vegetarian and vegan diets, and then carried out additional calculations to quantify nutritional quality of said diets. Hallström et al. (2015) examined 49 dietary scenarios generated from 14 studies in search of consumer-driven mitigation strategies for food-system environmental deterioration. Venturing beyond articles exclusively employing the LCA framework, Jones et al. (2016) employed a systematic survey of literature summarising wider sustainability assessments of diets, whilst Ridoutt et al. (2017) critically interpreted diet-level studies in line with the United Nations Sustainable Development Goals. González-García et al. (2018) compared the carbon footprints and nutritional quality of 66 daily diets sourced from 12 peer reviewed papers. Finally, Hallström et al. (2018) investigated the adoption and efficacy of nutrient profiling tool in an LCA context.

Collectively, these review articles clearly demonstrate a notable shift in attention from simpler mass-based LCA towards more nutritionally driven environmental assessments. Nevertheless, the adoption of such approaches is not without criticism. Hallström et al. (2018) raise concerns that nutrient profiling methods may not always be appropriate for diet-level assessments because many density scores were not necessarily designed for such use. Furthermore, with global meat consumption expected to increase for the foreseeable future (OECD-FAO 2018), dietary comparisons based on hypothetical scenarios, whilst useful to improve the evidence base of long-term strategies for sustainability, may not be the best methodological approach to make short to medium-term differences (Van Kernebeek et al. 2014). Without forced restrictions on supply chains, e.g. via carbon taxes on meat products (Briggs et al. 2013)—questionable strategies from a macroeconomic perspective (Jensen et al. 2015; Leslie 2018)—a mass-shift towards plant-only diets is unlikely and nutritionally contentious at a global scale, making such comparisons less relevant. Perhaps more importantly, diet-level LCA does not generate immediately actionable implications for food producers with regard to mitigation of their environmental footprints, as consumption patterns are largely beyond their control. Given that the vast majority of environmental burdens associated with agri-food systems physically originate from farms (Gerber et al. 2013), the lack of information as to how best to *produce* food significantly diminishes the potential of LCA studies to contribute to climate change mitigation.

Motivated by these current limitations of dietary LCA, this paper evaluates the strengths and weaknesses of recent literature on commodity-level LCA of agri-food systems, with the aim to identify potential pathways towards sustainability analysis that can inform not only consumers but also producers. The structure of the manuscript is laid out as follows: Sect. 2 details the inclusion criteria for existing studies and provides the main body of the literature review. Section 3 offers a critical interpretation to the current state of knowledge regarding the role of human nutrition in LCA-based environmental assessments. Finally, Sect. 4 concludes the paper with a brief summary of key findings and a discussion on pathways to further improvement.

## Review of single or multiple commodity studies which address nutritional composition

### Review selection criteria

For the purpose of initial screening, relevant literature was systematically sourced from *Scopus* using search terms “life cycle assessment” OR “carbon footprint” AND “nutrient density” OR “nutrition*.” The first 200 returns were considered for inclusion under the following criteria:

- Peer-reviewed journal articles
- Published after Heller et al. (2013), where the authors produced a summary of literature spanning 2003 to 2013
- Written in English
- Discusses environmental impacts as the primary focus; for example, epidemiological studies were excluded
- Employs functional units which address nutritional composition
- Primarily focuses on individual food or beverage commodities, whether on their own or as part of dietary scenarios

Using these criteria, 49 papers were identified for consideration. Of these, 27 were categorised as studies solely focusing on diet-level comparisons (e.g. vegan diet versus omnivorous diet) and subsequently excluded from the list, whilst 16 were classed as “product-level” and therefore included. In addition, six published review articles on nutrition-focused LCA, predominately at the diet level, were identified; these will be used in Sect. 3 to form a critical discussion to assess the current state of agri-food LCA research.

The review below is presented under three tiers of complexity in relation to how a study considers human nutrition in the LCA framework. Studies grouped into the first tier focus on single-issue functional units, such as environmental impacts per individual nutrient unit. The second tier employs composite indicators incorporating more than one nutrient, such as nutrient-profiling scores, as functional units. Finally, the third tier applies *commodity-level* nutritional values to *diet-level* analyses, combining production and consumption aspects of the issue into a single framework. Where a study combines more than one level of complexity, it is classified in the highest tier.

### Tier 1 approach: single-issue functional units

In a study of Breton pâté production, Teixeira et al. (2013) compared the carbon footprints of nine different production systems under mass-based (100 g of product), energy-based (kcal) and nutrition-based (protein) functional units. The systems were differentiated by farming practice (conventional, organic, *label rouge*, a French Governmental certification based on organoleptic properties determined by sensory panels, and *Bleu-Blanc-Coeur*, an initiative which promotes omega-3 fatty acid content through feeding regimes) and packaging types (tin can, aluminium can or glass jar). All pâté was produced from pigmeat. The system boundary was cradle-to-grave and included waste management at the end of the life cycle. The authors found that, on a mass basis, organic pâtés had higher carbon footprints than the other systems whilst conventional, *label rouge* and *bleu-blanc-coeur* products all had similar emission intensities. When considering the nutritional content, however, relative rankings were affected depending on which functional unit was adopted. For instance, when considering the carbon footprint in terms of g CO_2_-eq/g protein, the organic system performed marginally better than one of the conventional systems, due to a higher protein content driven primarily by the cuts of meat used in the pâté. On the other hand, energy-based carbon footprints considered as g CO_2_-eq/kcal suggested that the organic system once again fared least favourably, whilst relative rankings amongst the conventional and *bleu-blanc-coeur* systems varied to a certain degree, depending on the calorific content of individual pâtés. The authors concluded by stressing the importance of functional unit selection in comparisons of food products which generates a considerable effect on research findings.

Tyszler et al. (2014) proposed a framework to maximise information provided by single-issue functional units by utilising linear programming to create scenarios that replace individual food products with nutritionally equivalent alternatives. Using two weekly diet case studies as examples, the authors first replaced apples (*Malus pumila*) in the fruit component of a baseline diet—consisting of 3.6 servings of apples, 1.2 servings of oranges (*Citrus maxima × Citrus reticulata*), 1.2 servings of kiwis (*Actinidia deliciosa*) and 0.9 servings of strawberries (*Fragaria × ananassa*)—with an equivalent portion of oranges. As this change resulted in 4.8 servings of oranges and thus an excess intake of vitamin C, the authors used a constrained linear optimisation algorithm and removed the equivalent portions of kiwis and strawberries from the weekly diet. This substitution resulted in slightly higher carbon footprints compared with the baseline diet, energy requirements and land use. In the second case study, Tyszler et al. (2014) replaced 2.2 servings of chicken (*Galus galus domesticus*) and 0.8 servings of red meat with 3 servings of vegetarian burgers. Nutritionally, this replacement led to a deficiency in lysine, methionine and selenium. Under the constraint that livestock meat was excluded from the diet, the model then added 0.1 serving of salmon (*Salmo salar*) and 0.2 servings of cod (*Gadus morhua*) to meet the essential amino acids and selenium requirements. This time, the substitutions resulted in markedly lower carbon footprints, energy use and land use than the original diet. To circumvent the requirement for linear programming knowledge, the authors also developed a software package to allow other LCA researchers to perform similar studies. Despite the benefits of the approach, the authors point out that data requirements, in terms of nutritional quality and dietary habits, are highly intensive, which may restrict wider applicability.

In a comparison between conventional ultra-high temperature (UHT) milk and a nutritionally enhanced UHT milk in Spain, Roibás et al. (2016) used the LCA framework to consider carbon and water footprints of both production methods in combination with health effects. The authors used a cradle-to-gate system boundary with a baseline functional unit set as 1 l of packaged UHT milk leaving the dairy factory; nutritional values of the final products were calculated externally to the LCA framework. The enhanced UHT milk was produced through cow-feed supplementation of linseed (*Linum usitatissimum*), naturally high in omega-3 α-linolenic acid. As a result, it contained 1% less saturated fat but 82% more unsaturated fats, with the omega-6/omega-3 ratio dropping by 58% compared with the conventional UHT milk. The enhanced milk also had around three times more selenium than the conventional milk. Based on epidemiological studies, the authors asserted that improved conjugated linoleic acid, lower ratios of omega-6/omega-3 and higher levels of selenium could have benefits to human health in the form of reduced instances of atherosclerosis, certain types of cancer and contributions to normal thyroid and immune system functions. Regarding the environmental footprints, although the production of fodder in the enhanced system had higher greenhouse gas (GHG) emissions than the conventional fodder (mainly maize (*Zea mays*) and soybean (*Glycine max*) meal), methane emissions from manure management and enteric fermentation were lower in the enhanced system, making the total carbon footprint lower also. The water footprint, on the other hand, was marginally higher (2%) than the conventional system, but the authors concluded that when farm-level variation was included, no significant differences were drawn.

Motivated by differences in product-level supply of essential amino acids (EAA), Tessari et al. (2016) compared land use and carbon footprints of 15 foods (including beans (*Fabaceae*), cauliflower (*Brassica oleracea*), beef, fish, maize, milk, peas (*Pisum sativum*), potato (*Solanum tuberosum*), quinoa (*Chenopodium quinoa*) and rice (*Oryza sativa*)) under three different functional units. The baseline functional unit was arbitrarily chosen as 100 g edible fraction of each product. The second and third functional units, on the other hand, were determined by the mass of the product required to provide (1) 13 g of total EAA, irrespective of deficiencies in certain amino acids, and (2) recommended quantities of all individual EAA, regardless of oversupply of certain amino acids, respectively, for a 70 kg male. Data on land use and carbon footprints were sourced from previously published studies whilst nutritional composition was obtained from Italian national nutrient tables. Under the first functional unit of 100 g of edible product, meat, fish and peas had considerably higher demand for land use than the other foods whilst beef and fish tended to have the highest carbon footprints. Switching to EAA-specific functional units, however, resulted in marked rank reversals. For instance, beans, peas, potatoes and rice required substantially more land to provide a human with the recommended intake of all EAA in comparison with 100 g of edible product. Regarding carbon footprints, beef, cauliflower and rice demonstrated the greatest changes across functional units, with EAA-based estimation resulting in markedly lower GHG emissions relative to the 12 other products. The authors point out that, when detailed protein requirements are accounted for, environmental gaps between livestock products and vegetables can be notably reduced, reinforcing the argument that mass-based comparisons are often an inappropriate use of functional units.

Schaubroeck et al. (2018) conducted a sustainability scoring exercise for canteen meals offered at Ghent University, Belgium, using a functional unit of one meal regardless of its energy or nutritional content. Sustainability was assessed according to: ecological scoring, nutritional scoring, sustainability of suppliers and other information considered important by key stakeholders. Ecological scores were largely determined through LCA studies of the composite ingredients in each meal, or studies of entire meals themselves, and simplified to single score comparisons across meals based on a range of endpoint ecological footprint impact categories. Nutritional scores were based on meals’ provision of energy, protein, fat, saturated fat, carbohydrates, sugar and salt. These scores were expressed by integers based on the number of nutritional criteria met. Suppliers’ sustainability was assessed on readily available information such as adoption of water recycling. Lastly, additional information collected was determined based on a qualitative case study with producers and consumers, who identified issues such as the inclusion of genetically modified organisms in meals or consideration of animal welfare. Based on the information gathered from each meal, the authors suggested that meal providers could provide colour-coded indicators on posters or menus to indicate the level of sustainability under each of the four themes addressed. Schaubroeck et al. (2018) also highlighted the limited sustainability information provided by LCA studies due to the sole focus on environmental issues, which are not the primary aspect of decision-making for some consumers.

### Tier 2 approach: multiple nutrients within single functional units

Doran-Browne et al. (2015) applied the concept of nutrient density scores (NDS) in the assessment of GHG emissions attributable to agricultural products typically found in southeast Australian diets. Four functional units were considered: mass of product (t); mass of protein (t); energy content (GJ) and NDS. The NDS of each product was determined according to the Nutrient Rich Food model (NRF9.3) originally developed by Fulgoni et al. (2009), whereby higher contents of nine encouraged nutrients (protein, fibre, vitamins A, C and E, calcium, iron, magnesium and potassium) are associated with a higher score, and three discouraged nutrients (saturated fat, sodium and added sugar) with a lower score. The quantity of each nutrient present in a product was first divided by its recommended daily intake (RDI), or daily allowances (RDA) for discouraged nutrients, to obtain the percentage of RDI satisfied by the product, and subsequently converted to a weighted score according to the product’s relative importance as measured by energy value. The NDS for each product was then derived as the difference between the sum of these weighted scores associated with “positive” nutrients and the sum of the similar scores associated with “negative” nutrients. The food products assessed were beef (lean and untrimmed), lamb (lean and untrimmed), regular milk, reduced fat milk, wheat (*Triticum aestivum*) flour and canola (*Brassica napus*) oil. When using the standard mass metric (t CO_2_-eq/t product), the authors found that wheat flour generated the lowest GHG emissions whilst milk and canola oil had similar levels of impacts. Meat products had the highest impacts, with the lean cuts having a higher CO_2_-eq value than the untrimmed cuts. However, when the novel metric (t CO_2_-eq/NDS) was applied, the lean cuts had considerably lower environmental impacts than the untrimmed cuts, and the gap between non-meat products and lean meat was substantially narrowed. Similarly, both regular and low-fat milks were found to have considerably lower impacts than canola oil, whereas wheat flour still had the lowest impacts. Although not covering the whole life cycle of products and stopping short of distinguishing between different compounds within each nutrient group, for example between polyunsaturated (PUFA) and monounsaturated fatty acids (MUFA), and amongst different EAA, the study proposes a useful technique for comparing different food groups based on their nutritional value.

Using industrial data and national nutritional statistics from France, Drewnowski et al. (2015) explored interlinkages between carbon footprints and nutrient densities for 661 different foods and beverages. Carbon footprints were calculated under 100 g and 100 kcal functional units, with their relationship with NDS subsequently analysed using linear regression. Thirty-four food categories considered by the authors were split between five common food groups: meat and meat products, milk and dairy products, frozen and processed fruit and vegetables, cereals and sweets. Two density scores were calculated, with one accounting for six encouraged nutrients (ND-6; protein, potassium, magnesium, calcium, phosphorus and vitamin D) and the other accounting for 15 encouraged nutrients (ND-15: ND-6 nutrients plus fibre, vitamins A, C and E, iron, thiamine (vitamin B1), riboflavin (vitamin B2), niacin (vitamin B3) and folate (vitamin B9)). Grains and sweets were found to have the lowest carbon footprints of the major food groups regardless of functional unit but were also found to have low density scores. When reported per gramme, meat and meat products tended to have the highest carbon footprints across all food groups; when reported on an energy basis, however, processed and frozen fruit and vegetables leapfrogged meat and meat products and had the highest carbon footprints due to their low energy densities. The authors pointed out that nutrient-dense foods such as meat and dairy products typically have high carbon footprints, with the reverse also being true (foods with low nutrient density tend to have lower carbon footprints). Drewnowski et al. (2015) also demonstrated the complexity of choosing suitable functional units in comparative LCA of food products with marked reversals in relative rankings in system-wise environmental performance, and posited that to formulate a truly sustainable diet, simply focusing on one metric, carbon footprints in this instance, is not an effective assessment method.

In an attempt to identify a functional unit suitable for capturing a wider measurement of the sustainability of food products, Masset et al. (2015) examined environmental footprints of foods and drinks representative of a typical French diet under a number of different impact categories. The authors defined sustainable food products as low emitting, affordable and of high nutrient quality, determined collectively by a final score that takes the value: 0, 1, 2 or 3. Nutritional quality of food products was determined in one scenario using the French SAIN, LIM method, whereby five nutrients (protein, fibre, calcium, vitamin C and iron) are encouraged whilst three (saturated fat, added sugar and sodium) are discouraged. If a food product obtained more than 97% of its energy from fat (as is the case for nuts and oils), then vitamin E, MUFA and α-linolenic acid contents were also accounted for in the encouraged nutrient profile. A product’s overall sustainability score was then derived by comparing its performances in the aforementioned three areas against their respective median values; a product received a point if its GHG emissions and price were lower than the median, and if its nutritional score was higher than the median. Masset et al. (2015) argued that mass and energy-based functional units are generally unhelpful in determining sustainable products. In particular, they demonstrated how functional unit manipulation can affect relative rankings across food products, with those of monogastric meat products and fruits/vegetables easily reversed between energy-based and nutrition-based computations of GHG emissions.

Building upon the NRF9.3 framework described above, Saarinen et al. (2017) developed a novel nutrient index to specifically compare the overall quality of protein-rich foods. The authors used multiple functional units such as individual nutrients applied per mass of product (e.g. CO_2_-eq/g calcium or /μg cobalamin (vitamin B12)), as well as a novel nutrient score specifically designed for protein-rich foods, which included MUFA, PUFA and vitamins B2 and B9, but excluded nutrients that are not typically provided in abundance by these food groups (e.g. magnesium and potassium). Global warming potential (GWP) was then estimated under both mass-based and nutritional score-based denominators. Nutrient contents of individual products as well as recommended intake values were sourced from public databases in Finland, whilst background LCA data were gathered through published literature. Twenty-nine food products ranging from cereals and pulses to dairy products, meat and seafood were considered. Saarinen et al. (2017) demonstrated that the choice of functional unit can affect interpretation of results considerably. For example, beef had the largest GWP on a mass-based functional unit (100 g of product) but overtaken by cheese and lamb as the most burdensome food group when the functional unit was changed to the nutrient content included in 100 g of product. In general, animal-based products had higher environmental impacts than cereals and pulses regardless of the functional unit, although the authors did not consider contents of some important micronutrients such as vitamin B12 or account for bioavailability of nutrients (e.g. haem iron) when consumed in different forms of food.

In a study focusing on replacing refined wheat flour used to produce typically cereal-based products with Canadian yellow pea (*Lathyrus aphaca*), Chaudhary et al. (2018) examined the effects on nutritional and environmental performance. Three common products were considered: pan bread, breakfast cereal and pasta; and three functional units were considered: kg food, single serving and nutrient density in a single serving. Nutritional content was assessed according to a nutrient balance concept which consists of three metrics similar to those proposed by Fulgoni et al. (2009): qualifying nutrients; disqualifying nutrients and the nutrient balance score (NBS). The qualifying nutrients were made up of 27 micro- and macronutrients including a wide range of minerals and vitamins as well as water, fibre, protein, α-linolenic acid and linoleic acid. The disqualifying nutrients were sugar, sodium, total fat, saturated fat and cholesterol. The three products were assessed according to Canadian recommended intake values (for qualifying nutrients) and maximum intake values (for disqualifying nutrients) under both typical ingredients and systems which included yellow pea. Carbon footprints were calculated where possible using Canadian-specific data; where this was impossible, EU-specific data were sourced from inventory databases. Nutritional values and mass-based carbon footprints were combined by dividing the NBS by the carbon footprint of each product related to typical serving sizes (75 g bread, 30 g breakfast cereal and 85 g pasta). The authors found that replacing refined wheat flour with yellow pea flour improved the NBS by 11, 70 and 18% for bread, breakfast cereals and pasta, respectively, whilst simultaneously reducing carbon footprints of 1 kg of each product by 4, 11 and 13%. Chaudhary et al. (2018) concluded that pulses could play a pivotal role in improving human nutrition and reducing the food sector’s carbon footprint in line with the United Nations’ Sustainable Development Goals.

Modifying the framework devised by Saarinen et al. (2017), McAuliffe et al. (2018a) explored the effect of adopting nutritional functional units on relative rankings amongst the most common meats consumed in the UK (beef, chicken, lamb and pork). Using previously published carbon footprint and carcass yield data, the authors first compared carbon footprints of products based on mass functional units (100 g of deboned meat), and then examined effects of adopting nutritional functional units based on omega-3 fatty acid content and NDS. System boundaries were set as cradle to farm gate, and where appropriate, liveweight was converted to carcase weight and subsequently to meat ready for cooking. Secondary processing (e.g. pork made into sausages) was not considered. Data from nutritional tables were used to develop UK-specific NDS based on scores produced by Saarinen et al. (2017) as well as a novel NDS which included zinc, selenium and vitamin B12. The authors reported that ruminant systems tended to have higher environmental impacts than their monogastric counterparts when compared on a mass basis. However, when the nutritional composition of meats was included in the carbon footprint analysis, emissions attributable to beef production, particularly from concentrate-finished systems, were found to be comparable or lower than pig and chicken production depending on management practices. The authors concluded that nutrient density of products provides important information to improve environmental performances of agri-food systems; equally, they observed that eating smaller portions of higher quality products may improve human nutrition whilst simultaneously reducing the global carbon footprint through potentially decreased food production.

Xu et al. (2018) analysed a wide range of common local sources of carbohydrates in China. The authors used several functional units to examine differences between products; these consisted of mass, energy, protein, carbohydrate and two nutrient profile scores. From an environmental perspective, carbon footprints were calculated using China-specific data where possible. Nineteen products were evaluated in total, grouped into five categories of rice, wheat, maize, potato and pulses. The two nutrient profiles consisted of 11 (NU11: protein, carbohydrate, fibre, vitamins B1, B2 and B3, calcium, iron, zinc, magnesium and selenium) and 21 (NU21: all of the above plus fat, vitamins A, C and E, potassium, sodium, manganese, copper, phosphorus and cholesterol) nutrients, respectively, and were calculated similarly to formulae set out in Fulgoni et al. (2009). Regardless of the functional unit, rice generated the highest carbon footprint due to large methane emissions resulting from anaerobic conditions. However, there were notable reversals to relative rankings amongst other products. For example, when compared on a mass basis, potatoes tended to have the lowest carbon footprints, but when the functional unit was switched to protein and NU11, the carbon footprint of potatoes became larger than maize kernel and sorghum (*Sorghum bicolor*) flour, respectively. The authors concluded that food comparisons should always account for the nutritional composition of individual products and pointed out the opportunity for China to considerably reduce its agricultural emissions by switching from rice-based diets to those supported by other sources of carbohydrates.

Hallström et al. (2019) investigated seafood products that should be recommended to consumers based on a combined assessment of nutritional qualities and environmental performances. Acknowledging the sensitivity of NDS to its mathematical formulation, the authors calculated seven different NDS for 37 Nordic commodities. Following a detailed comparison of NDS, Hallström et al. (2019) concluded that the scoring method most appropriate for their goal was the 24-nutrient system, which incorporate the densities of 22 qualifying nutrients and two disqualifying nutrients. A mass-based reference flow (100 g edible product) was deemed sufficient as the base (denominator) of NDS calculation, as energy and water content of seafood products did not vary enough to warrant the inclusion in the formula. Despite the general perception that all fresh seafood is nutritious and climate-friendly, 37 products were found to differ considerably both in terms of environmental and nutritional performance; certain species such as shrimp (*Pandalus borealis*) and plaice (*Pleuronectes platessa*) scored significantly less overall than pelagic species such as herring (*Clupea harengers*) and mackerel (*Scomber scombrus*). The authors note, however, that further work is required to consider other environmental impacts of seafood production systems, for example toxins arising from water-based pollutants such as micro-plastics and mercury.

### Tier 3 approach: commodity-level scores incorporated into diet-level analysis

Stylianou et al. (2016) developed the Combined Nutritional and Environmental Life Cycle Assessment (CONE-LCA) method to empirically apply the conceptual framework devised by Heller et al. (2013). CONE-LCA is a hybrid approach that utilises traditional midpoint LCA modelling but adjusts output values to predict endpoint metrics based on the nutritional quality of food products. The ultimate output of this novel approach is the impact of diet change not only on midpoint environmental measures (GWP and respiratory effects via particulate matter in this particular case) but also the human health effects of these impact categories represented as disability-adjusted life years (DALY). The authors carried out a case study whereby an extra serving of milk (244 g), acting as a de facto functional unit, was added to three dietary scenarios: no changes to the rest of the diet; removal of other food products with an equal caloric value (119 kcal), and removal of an equal caloric quantity of sugar sweetened beverages. The authors used epidemiological data to assess milk’s effects on human health, both positive and negative, as expressed by DALY. Whilst adding a serving of milk to the diet increased both GWP and respiratory inorganics at the midpoint, milk consumption was found to be beneficial for long-term health, as reduced risk of colorectal cancer and stroke outweighed increased risk in prostate cancer in all scenarios. Whilst Stylianou et al. (2016) acknowledge that uncertainty associated with endpoint impact assessments is considerable, their framework is a significant contribution to the methodological advancement of nutrition-based LCA.

Sonesson et al. (2017) developed a new index to account for differences in protein quality between food products under the LCA framework. They established a new functional unit, protein quality index-adjusted mass, for each food product studied (bread, chicken breast, minced pork, minced beef, milk and pea soup) based on contents of the nine EAA in that particular product as well as in the overall diet. Protein quality index was formulated in such a manner that, if a particular EAA was deemed deficient in a given diet, food products with higher contents of the said EAA scored higher (and vice versa). The performance of each food product was then evaluated within the context of three diets: an average Swedish diet; a lacto-ovo vegetarian diet; and a low meat diet. Under the average Swedish diet, meat products, particularly beef, scored poorly and, as a result, were judged to have high marginal environmental impacts. Conversely, under the low meat diet in which EAA such as leucine and lysine tend to be deficient, meat products scored favourably, and the results subsequently reversed. Through this example, the authors showcased the importance of considering the nutritional value of each food group in a wider picture of human dietary requirements and food availability.

Building upon the aforementioned protein-focused work published in 2017, Sonesson et al. (2019) extended their nutrition-in-a-dietary-context method to 12 micro- and macronutrients by employing the NRF9.3 profiling score as a functional unit. In this study, the authors focused on carbon footprints of seven food products: bread, apples, tomatoes (*Solanum lycopersicum*), milk, hard cheese, spread and chicken fillets. Echoing the argument from their 2017 paper, the authors posited that nutrient scores are only meaningful if they are accounted for in the context of an individual’s diet; therefore, they considered NDS applied to an average Swedish diet and a typical “unhealthy” diet. The diets were first differentiated by contents of individual nutrients, with the unhealthy diet shown to contain considerably more sodium, sugar and saturated fat than the average Swedish diet. Then, for each nutrient, the diet-level ratio between total content and RDI was used to determine context-specific commodity-level density scores. Using bread as the baseline product for comparison, the authors observed that apples and tomatoes had lower GWP whilst all other products had higher GWP when compared on a mass basis. When considering marginal nutrient requirements in the average diet, however, the gap between bread and other products was narrowed considerably. For example, in the mass-based comparison, chicken fillets had a GWP approximately six times higher than bread; whereas, when nutrient supply was accounted for, this difference reduced to approximately two times higher due to the higher nutrient density of chicken. In the unhealthy diet, the differences remained at around six times higher for chicken due to the relative oversupply of certain nutrients, in particular saturated fat.

## Discussion

### Effects of farm management on commodity-level nutrition

As discussed in Section 1, one of the benefits of commodity-based LCA studies is that their outputs provide clearer guidelines for immediate actions by farmers. It is widely known that farming strategies influence product quality and nutrition. For example, grass (*Lolium* spp.) and clover (*Trifolium* spp.) contain higher levels of omega-3 fatty acids than cereals (Moorby et al. 2009), resulting in pasture-based ruminants producing meat (Warren et al. 2008) and milk (Ferlay et al. 2006) with higher levels of beneficial fats than those fed typical cereal (concentrate) rations. In addition to the effects of pasture or forage silages on nutrition, recent research has shown that including linseed (Skiba et al. 2015) or algae (Lemahieu et al. 2013) in monogastric livestock diets can improve fatty acid profiles of pigmeat and poultry products. Interest in this technological advancement is evident with the rise of naturally enriched omega-3 chicken across European supermarkets and also the *Bleu-Blanc-Coeur* initiative in France, of which environmental implications (Teixeira et al. 2013) were described earlier. Nutritional quality of meat is also highly dependent on the section of the animal in question, with fattier areas such as ribs tending to be lower in protein than leaner cuts such as fillet, and offal being significantly higher in certain vital nutrients: liver, for example, is a rich source of vitamins A, B2, B9 and B12 as well as iron, copper and choline. Plant nutrition is also highly dependent on factors regulating growing conditions such as soil organic matter (Wood et al. 2018), soil pH which affects the ultimate bioavailability of nutrients (Spadoni et al. 2007), “geo-nutrition” or nutrient content of the soil (Joy et al. 2014) and plant genetics (Zuo and Zhang 2011). By design, nutritionally focused LCA studies have a capability to address these issues more effectively than mass-based studies.

Such potential, however, has not yet been fully realised in literature, even at the commodity level. Table 1 shows that only six of the 16 studies reviewed in Sect. 2 consider different farm management strategies, defined by consideration of two or more agricultural practices for the same crop (e.g. organic vs. conventional) or livestock (e.g. pasture vs. feedlot). The remaining ten studies do not account for differing farming practices, although some include multiple processing practices (Chaudhary et al. 2018) and supply chain management strategies (Schaubroeck et al. 2018). One example to demonstrate the broken linkage between farming and food consumption is Teixeira et al. (2013), who investigated the environmental impacts of four pig farming systems and how much protein would ultimately end up in the final pâté. Despite one of their farming scenarios being the *Bleu-Blanc-Coeur* initiative with omega-3 enhanced feed, the authors did not consider fatty acids as part of their nutritional analysis. Doran-Browne et al. (2015) analysed environmental consequences of different farming systems, different cuts of meat (lean or untrimmed) and different types of milk (whole-fat and skimmed) as separate comparisons. However, the authors stopped short of including the causal effects brought about by different farming systems on nutritional quality of the final product. Roibás et al. (2016) explored the environmental impacts of various UHT milk products and directly accounted for product quality in terms of both long-chain and short-chain fatty acids contents from each system. Even though the authors opted not to calculate NDS for each of the products, they did consider individual nutrients which Western populations tend to be deficient in (i.e. some long chain PUFA and selenium). McAuliffe et al. (2018a) examined different production practices for commonly consumed meats in the UK, accounting for nutritional quality from pasture- or concentrate-finished beef cattle, upland or lowland lamb and free-range or organic chicken. A major drawback to this approach, however, is that the authors relied on meat science data and environmental footprints sourced from comparable but unrelated secondary material; therefore, the hypothetical systems created were not directly linked from farm to fork. Finally, it is evident that none of the studies considered plant growing conditions, many of which would affect the nutritional quality of food and feed as already described.

**Table 1:** Summary of attributes specific to each of the included studies

Study	Primary product(s)	Study region	Functional units	Number of nutrients (maximum)	Nutrient density included	System boundary	Impact categories	Farm-level management included	Complexity tier^a^
Teixeira et al. (2013)	Pâté	France	100 g pâté, 1 kcal, 1 g protein	1	No	Cradle to cradle (full life cycle)	GWP	Yes	1
Tyszler et al. (2014)	Apples, oranges, chicken, beef, vegetarian burgers, cod, salmon	Netherlands	100 g, 1 portion	36	No	NS	GWP; EU; LU	No	1
Doran-Browne et al. (2015)	Lamb (with and without fat), beef (with and without fat), whole milk, skimmed milk, wheat flour, rapeseed oil	Australia	1% nutrient density, t product, t protein, GJ energy	12	Yes	Cradle to processing (prior to packaging)	GWP	Yes	2
Drewnowski et al. (2015)	483 foods and beverages	France	100 g, 100 kcal, nutrient densities correlated with mass or energy	15	Yes	Cradle to retail	GWP	No	2
Masset et al. (2015)	373 foods	France	100 g, 100 kcal, nutrient densities correlated with mass or energy	8	No	NS	GWP	No	2
Roibás et al. (2016)	Conventional UHT milk; enhanced UHT milk	Spain	1 l packaged milk, 55 μ Se/day	1	No	Cradle to dairy factory exit	GWP; WF	Yes	1
Tessari et al. (2016)	15 common food products	Italy	100 g edible product, 13 g total essential amino acids, edible mass required to supply all essential amino acids	9 (essential amino acids)	Yes^b^	NS	GWP; LU	No	1
Stylianou et al. (2016)	Weighted average of whole, skimmed, semi-skimmed and non-fat milk	USA	1 serving of milk (added to a diet or replacing other calorific sources)	NA	No	NS	HHD; EQ; RU; ES	No	3
Saarinen et al. (2017)	29 common Finnish foods	Finland	100 g, 6 nutrient density scores	12	Yes	Cradle to consumption	GWP	No	2
Sonesson et al. (2017)	Bread, chicken fillet, minced pork, minced beef, milk, pea soup	Sweden	1 kg product, 1 g protein, 1 g digestible protein, protein quality index	9 (essential amino acids)	Yes^b^	Cradle to preparation for consumption	GWP	No	3
Chaudhary et al. (2018)	Yellow pea, bread, breakfast cereals, pasta	Canada	Nutrition carbon footprint score (nutrient balance/carbon footprint per serving)	27	Yes	Cradle to food manufacturing	GWP	No	2
McAuliffe et al. (2018a)	Beef, chicken, lamb, pork	UK	100 g meat, 1 g omega-3 fatty acids, 1 g non-competing omega-3 fatty acids, 1% nutrient density score	13	Yes	Cradle to farm gate	GWP	Yes	2
Schaubroeck et al. (2018)	42 canteen meals	Belgium	1 meal serving	7	No	Cradle to food preparation at canteen	EF	No	1
Xu et al. (2018)	19 carbohydrate-rich foods grouped as: rice, wheat, potato, maize, pulses	China	1 kg product, carbohydrates/kg product, protein/kg product, energy/kg product, 2 nutrient profiles scores	21	Yes	Cradle to processing (prior to retail)	GWP	Yes	2
Hallström et al. (2019)	37 types of seafood	Sweden	Seven different nutrient density scores	24	Yes	Cradle to fish landing	GWP	Yes	2
Sonesson et al. (2019)	Bread, apples, tomatoes, milk, hard cheese, spread, chicken fillets	Sweden	Nutrient density score, nutrient score in relation to a dietary context	12	Yes	Cradle to processing	GWP	No	3

*EF*, ecological footprint; *EQ*, ecosystem quality; *ES*, ecosystem services; *EU*, energy use; *GWP*, global warming potential; *HHD*, human health damage; *LU*, land use; *RU*, resource use; *WF*, water footprint

^a^Complexity tiers are defined as: (1) solely relying on single-issue functional units, (2) considering multiple nutrients within single functional units and (3) utilising commodity-level nutritional information in diet-level assessments

^b^These studies adopt amino acid density, as opposed nutrient density with a wider range of micro- and macronutrients, to evaluate the nutritional value of products

As farming practices are known to affect the flow of nutrients from soil to the end-product regardless of whether plant based or animal based, the general lack of consideration to these factors in current literature leaves a knowledge gap which requires attention. Ideally, studies which compare food items should account for farming practices and resulting nutritional compositions of the end-product; however, this level of analysis demands detailed supply chain information from cradle to processing (or cradle to plate if cooking is included). Data requirements to achieve this are inevitably vast, which will likely be a limiting factor in the short-term. As information availability becomes deeper and life cycle inventories more robust, the feasibility of such studies should improve; in the meantime, environmental comparisons of edible products fail to accurately address the function of food as a source of human sustenance when they do not account for nutritional value in any manner. This also results in reduced information value from the producer’s perspective.

### Role of diet-level LCA

In line with the scope of this article outlined in Sect. 1, the studies reviewed herein have specific focus on individual food products. As discussed, however, the majority of nutritional LCA work to date has centred on dietary comparisons; 27 studies identified under the defined search criteria were classified as diet-level LCA, almost twice the number of studies addressing individual commodities. Nonetheless, it is acknowledged that, when examining the complexities of individual studies in greater detail, the line between diet-level LCA and commodity-level LCA sometimes becomes blurred. To effectively identify novel opportunities to create more sustainable agri-food systems, it is therefore worth overviewing reviews of diet-level LCA studies published elsewhere in literature.

These six reviews (see Sect. 2.1) have collectively recognised key strengths and constraints of dietary comparisons in an environmental context. Naturally, one of the key benefits is providing an environmentally interesting answer to “what would happen if everybody ate *Diet A* rather than *Diet B*”, but, arguably, this is also the central weakness. For example, diets are often hypothetically created by authors via secondary material and, therefore, do not reflect true consumption patterns (Van Kernebeek et al. 2014). Indeed, it has been suggested that hypothetical plant-based diets tend to have significantly lower environmental burdens than those based on recorded consumption patterns (Hallström et al. 2015, Vieux et al. 2013), indicating that such comparisons should be interpreted with caution. Jones et al. (2016) bolster this by arguing that dietary scenarios need to reflect the dynamics of consumer behaviour by tailoring methodological frameworks to include such fluidities. Although it would be impractical for LCA experts to carry out consumption surveys as standard, authors could rely more on consumers’ revealed preference rather than “textbook” recommendations which are rarely adhered to by the general population. Despite these limitations, diet-level LCA comparisons unequivocally reach a similar conclusion: a reduction of meat consumption in stereotypically Western diets will generally improve environmental performances of food systems (González-García et al. 2018). As already discussed, however, propositions that require dietary changes cannot provide a prompt solution, particularly those based on drastic scenarios.

The choice between product-level and diet-level investigation is based entirely on the goal and scope of a study, and as discussed, there are notable drawbacks for each of the approaches. In addition to data availability issues discussed in Sect. 3.1, product-level LCA often fails to account for nutritional complexities such as bioavailability and individual dietary requirements—as is the case in a recent large-scale meta-analysis by Poore and Nemecek (2018). Nonetheless, a combination of both product-level and diet-level methods has shown encouraging results in recent studies. In particular, Sonesson et al. (2017, 2019) devised a framework to calculate how much of a product would be required to satisfy EAA requirements (2017) and wider macro- and micronutrient requirements (2019) under different dietary scenarios, providing a suitable platform to elucidate environmental and nutritional performance for various stakeholders across multiple supply chains. The authors acknowledge that their framework is in its infancy and currently suffers from some methodological drawbacks such as a lack of framework for uncertainty analysis. Notwithstanding, this novel hybridisation of two approaches is a useful reconciliation to minimise collective methodological weaknesses.

### Pathways to informative agri-food LCA to consumers and producers

#### Selection of functional units

All 16 studies included in this review contributed to the evolution of functional units in one way or another. It should be noted, however, that the term “functional unit” raises some ambiguity in agri-food systems because, depending on the stakeholder, this could mean different things. For instance, in the case of farmers who grow crops for their livelihood, the function of their farm is to generate revenue which is determined largely by yield (and market conditions outside of their control). Therefore, the most relevant functional unit for an arable farmer may be yield per hectare or profit per hectare. Across arable LCA literature, the functional unit of yield per hectare is often adopted (Esteves et al. 2016) in parallel with liveweight (Basset-Mens et al. 2006), liveweight gain (McAuliffe et al. 2018b) or carcase weight (Mogensen et al. 2015) in livestock LCA; yet, profitability of farming systems is rarely accounted for leaving room for more detailed assessments of economic-environmental trade-offs. As background inventories such as *ecoinvent* (Wernet et al. 2016) and Agri-footprint (Durlinger et al. 2017) have become more sophisticated and computing power grown exponentially, LCA capabilities have simultaneously improved to adopt wider system boundaries within agri-food studies. In combination with growing consumer awareness of environmental issues (Jaca et al. 2018), these capabilities have shifted the focus of many studies from farmers to consumers, resulting in the conundrum that agricultural yields have little “function” other than computationally convenient reference flows within a wider system. If consumers are to be considered the key stakeholders in an LCA study, then the main function of food is to provide nutrition. The studies included in the current review have addressed nutrition and health in multiple ways (Table 1), paving the way to link farmers to consumers once again.

#### Selection of nutrient profiling methods

Hallström et al. (2018) point out that nutrient profiling, whilst an undeniably useful method to address nutritional complexities inherent in food systems, is often used and interpreted incorrectly in environmental assessments. Although as many as 10 out of the 16 papers reviewed here considered nutrient density in one form or another (Table 1), many of them base their method on Fulgoni et al. (2009), which recommends inclusion of 9 encouraged nutrients and 3 discouraged nutrients into the nutrition score, without providing justifications for its selection. The 9.3 assumption, however, results in omission of essential micronutrients such as vitamin B12 and selenium, and as a result tends to favour plant-based products to comparable products of animal origin. This approach and its derivatives, therefore, are not necessarily a suitable method for comparing, say, fruits/vegetables with meat, although it is actively used in this manner (Doran-Browne et al. 2015; Drewnowski et al. 2015; Sonesson et al. 2019). A simple solution to this issue is to constrain comparisons with commodities within a single food group that forms part of an “eatwell” plate (Public Health England 2016) or to include as many nutrients as feasibly possible into a single index number (Chaudhary et al. 2018; Xu et al. 2018); however, the former approach diminishes the study’s information value for consumers whilst the latter amplifies the related but separate complexity surrounding weighting—as not all nutrients are equally beneficial to human health. More robust solutions are thus required: one such approach, as described in Sect. 3.3, is to compare multiple diets in the context of an over/under supply of particular nutrients (Sonesson et al. 2017, 2019). Albeit a data-intense approach, this currently seems to be the best proposed method to transparently and quantitatively assess nutrient density in an LCA framework. When data limitations make this procedure infeasible, sensitivity analyses of the effect of NDS choice, as carried out by Hallström et al. (2019), provides a realistic alternative.

#### Selection of impact categories

All papers selected for this article accounted for GWP either directly (14 studies) or indirectly (Stylianou et al. 2016; Schaubroeck et al. 2018), whereas only five studies considered alternative impact categories (Table 1). Whilst this is perhaps unsurprising given the global significance of climate change, it equally demonstrates a lack of attention being bestowed upon other mid-point environmental indicators such as eutrophication and acidification or end-point ecological impacts such as biodiversity losses. Furthermore, the five studies with non-GWP estimates are mutually incomparable due to a lack of overlapping impact categories. For example, Roibás et al. (2016) included a water footprint which is an important consideration under Mediterranean climate but less critical in temperate maritime conditions typically found in northern Europe. Tyszler et al. (2014) calculated demands on energy use and land use, but none of the other studies do the same. Stylianou et al. (2016) focused on endpoint impact categories, which, although essential to methodological development, restrict interstudy comparability primarily due to data limitations. The latter issue has since been partly mitigated by the halfway approach known as the DALY Nutritional Index (DANI) scoring of foods and diets (Weidema and Stylianou 2019); as the authors themselves concede, however, estimating health impacts of food consumption involves a very high level of uncertainty. It is noteworthy that goals and scopes, and thus system boundaries, across studies are also inconsistent. For instance, McAuliffe et al. (2018a) consider the nutrition of raw meat converted from liveweight at the farm gate, whilst Saarinen et al. (2017) include processing, retail and cooking. These observations reiterate the issue of limited inter-study comparability raised previously (McAuliffe et al. 2016), particularly when novel functional units and system boundaries are brought into an analysis.

In addition to insufficient examination of non-climate environmental impacts, a lack of consideration to the social pillar of sustainability has been identified as a major roadblock to further evolution of agri-food LCA (Nemecek et al. 2016). Social sustainability includes a wide range of concepts such as animal welfare (Scherer et al. 2018; Velarde et al. 2015) and noise pollution (Oltean-Dumbrava et al. 2016; Rodrigues 2018); however, the studies reported in this review collectively make a significant effort to address human nutrition, without a doubt a major component of this pillar. Dietary nutrition has direct and indirect effects, respectively, to quality of life (Schünemann et al. 2010) and health services (Stratton 2007). Likewise, the environmental quality of potable water (Pimentel 2009) and air pollution (Oakes et al. 2014) also affects human wellbeing. With air pollution in mind, Stylianou et al. (2016) combined environmental and nutritional indicators to establish a framework to account for both simultaneously. Sonesson et al. (2017, 2019) focused on the supply of nutrients to different diets reflecting alternative consumption patterns commonly found in Western demographics, whilst Schaubroeck et al. (2018) developed a novel approach to assess a wide range of sustainability metrics including socially important aspects such as the inclusion of GMOs. Despite a limited assessment of wider social sustainability in LCA literature, these particular articles demonstrate a marked step towards acknowledging that environmental burdens are not the only impacts associated with food systems which require attention.

#### Selection of communication strategies

Given the current state of rapid information spreading, accurate or otherwise, it is critically important that LCA studies are communicated clearly and transparently. Once a peer-reviewed paper is published, however, many LCA researchers are largely unaware of the usage of their work by policymakers and the wider public. The *LCA Food* biannual international conference is one of the rare occasions where academics, industry representatives and policymakers can meet and discuss the real-world applications of research outputs, and it has seen a large increase in the number of nutritionally-focused papers being presented in recent years (Nemecek et al. 2016). This does not only ensure that key policymakers are, at the very least, aware of a shifting consensus that nutrition and the environment are inextricably linked but also that researchers have regular opportunities to examine whether or not their work is communicated and interpreted accurately. Regarding communication with consumers, Schaubroeck et al. (2018) highlight how multidisciplinary work can aid purchase decisions using a “traffic light” system for various issues including ecological footprints, supplier sustainability and nutritional quality, making LCA results more accessible for laypeople. Finally, as a rare example of LCA studies nominating agricultural producers as key stakeholders, McAuliffe et al. (2018a) reveal how different production systems, for example grass-fed or concentrate-fed beef value chains, can affect the nutritional quality of the end-product. Effective tools and key messages such as these examples provide opportunities for the public to receive straightforward quantitative information which inherently acknowledges that mass-based product-level comparisons do not provide the complete picture.

## Conclusions

Food-oriented LCA studies are increasingly considering functional units which account for the nutritional composition of individual products and diets. The present paper has summarised how authors have recently addressed this transition from mass-based to quality-based model structures, with a particular focus on commodity-level analyses. Across a wide spectrum of studies, such a shift has been achieved through the inclusion of NDS (Doran-Browne et al. 2015) or, less frequently, consideration of individual nutrients such as selenium (Robias et al. 2016), omega-3 fatty acids (McAuliffe et al. 2018a) and vitamins (Saarinen et al. 2017). Other authors have added additional complexity by accounting for simultaneous impacts to the environment and human health (Stylaniou et al. 2016) and a product’s marginal contribution to the nutritional values of diets (Sonesson et al. 2017, 2019). The latter approach appears to be a successful first step in bridging the gap between diet-level and product-level LCA studies and providing implementable action plans for both consumers and producers. Even when this is infeasible, future research comparing multiple food categories or multiple production systems should at least acknowledge differences in nutritional composition and bioavailability between the final products and, ideally, the effects of these nutrients on overall dietary quality.

## References

[CR1] Basset-Mens C, Van Der Werf HMG, Durand P, Leterme P (2006). Implications of uncertainty and variability in the life cycle assessment of pig production systems. Int J Life Cycle Assess.

[CR2] Briggs ADM, Kehlbacher A, Tiffin R, Garnett T, Rayner M, Scarborough P (2013). Assessing the impact on chronic disease of incorporating the societal cost of greenhouse gases into the price of food: an econometric and comparative risk assessment modelling study. BMJ Open.

[CR3] Chaudhary A, Marinangeli C, Tremorin D, Mathys A (2018). Nutritional combined greenhouse gas life cycle analysis for incorporating Canadian yellow pea into cereal-based food products. Nutrients.

[CR4] de Vries M, de Boer IJM (2010). Comparing environmental impacts for livestock products: a review of life cycle assessments. Livest Sci.

[CR5] de Vries M, van Middelaar CE, de Boer IJM (2015). Comparing environmental impacts of beef production systems: a review of life cycle assessments. Livest Sci.

[CR6] Doran-Browne NA, Eckard RJ, Behrendt R, Kingwell RS (2015). Nutrient density as a metric for comparing greenhouse gas emissions from food production. Clim Chang.

[CR7] Drewnowski A, Rehm CD, Martin A, Verger EO, Voinnesson M, Imbert P (2015). Energy and nutrient density of foods in relation to their carbon footprint. Am J Clin Nutr.

[CR8] Durlinger B, Koukouna E, Broekema R, Paassen MV, Scholten J (2017) Agri-footprint 3.0. Gouda: Blonk Consultants

[CR9] Esteves VPP, Esteves EMM, Bungenstab DJ, dos Santos Wendriner Loebmann DG, de Castro Victoria D, Vicente LE, de Queiroz Fernandes Araújo O, do Rosário Vaz Morgado C (2016). Land use change (LUC) analysis and life cycle assessment (LCA) of Brazilian soybean biodiesel. Clean Technol Environ.

[CR10] Ferlay A, Martin B, Pradel P, Coulon JB, Chilliard Y (2006). Influence of grass-based diets on milk fatty acid composition and milk lipolytic system in Tarentaise and Montbéliarde cow breeds. J Dairy Sci.

[CR11] Fulgoni VL, Keast DR, Drewnowski A (2009). Development and validation of the nutrient-rich foods index: a tool to measure nutritional quality of foods. J Nutr.

[CR12] Gerber PJ, Steinfeld H, Henderson B, Mottet A, Opio C, Dijkman J, Falcucci A, Tempio G (2013) Tackling climate change through livestock—a global assessment of emissions and mitigation opportunities. Rome: Food and Agriculture Organization of the United Nations (FAO)

[CR13] González-García S, Esteve-Llorens X, Moreira MT, Feijoo G (2018). Carbon footprint and nutritional quality of different human dietary choices. Sci Total Environ.

[CR14] Hallström E, Carlsson-Kanyama A, Börjesson P (2015). Environmental impact of dietary change: a systematic review. J Clean Prod.

[CR15] Hallström E, Davis J, Woodhouse A, Sonesson U (2018). Using dietary quality scores to assess sustainability of food products and human diets: a systematic review. Ecol Indic.

[CR16] Hallström E, Bergman K, Mifflin K, Parker R, Tyedmers P, Troell M, Ziegler F (2019). Combined climate and nutritional performance of seafoods. J Clean Prod.

[CR17] Heller MC, Keoleian GA (2003). Assessing the sustainability of the US food system: a life cycle perspective. Agric Syst.

[CR18] Heller MC, Keoleian GA, Willett WC (2013). Toward a life cycle-based, diet-level framework for food environmental impact and nutritional quality assessment: a critical review. Environ Sci Technol.

[CR19] Jaca C, Prieto-Sandoval V, Psomas EL, Ormazabal M (2018). What should consumer organizations do to drive environmental sustainability?. J Clean Prod.

[CR20] Jensen S, Mohlin K, Pittel K, Sterner T (2015). An introduction to the Green paradox: the unintended consequences of climate policies. Rev Environ Econ Policy.

[CR21] Jones AD, Hoey L, Blesh J, Miller L, Green A, Shapiro LF (2016). A systematic review of the measurement of sustainable diets. Adv Nutr.

[CR22] Joy EJM, Ander EL, Young SD, Black CR, Watts MJ, Chilimba ADC, Chilima B, Siyame EWP, Kalimbira AA, Hurst R, Fairweather-Tait SJ, Stein AJ, Gibson RS, White PJ, Broadley MR (2014). Dietary mineral supplies in Africa. Physiol Plantarum.

[CR23] Lemahieu C, Bruneel C, Termote-Verhalle R, Muylaert K, Buyse J, Foubert I (2013). Impact of feed supplementation with different omega-3 rich microalgae species on enrichment of eggs of laying hens. Food Chem.

[CR24] Leslie G (2018). Tax induced emissions? Estimating short-run emission impacts from carbon taxation under different market structures. J Public Econ.

[CR25] Masset G, Vieux F, Darmon N (2015). Which functional unit to identify sustainable foods?. Public Health Nutr.

[CR26] McAuliffe GA, Chapman DV, Sage CL (2016). A thematic review of life cycle assessment (LCA) applied to pig production. Environ Impact Assess.

[CR27] McAuliffe GA, Takahashi T, Lee MRF (2018). Framework for life cycle assessment of livestock production systems to account for the nutritional quality of final products. Food Energy Secur.

[CR28] McAuliffe GA, Takahashi T, Orr RJ, Harris P, Lee MRF (2018). Distributions of emissions intensity for individual beef cattle reared on pasture-based production systems. J Clean Prod.

[CR29] Mogensen L, Kristensen T, Nielsen NI, Spleth P, Henriksson M, Swensson C, Hessle A, Vestergaard M (2015). Greenhouse gas emissions from beef production systems in Denmark and Sweden. Livest Sci.

[CR30] Moorby JM, Lee MRF, Davies DR, Kim EJ, Nute GR, Ellis NM, Scollan ND (2009). Assessment of dietary ratios of red clover and grass silages on milk production and milk quality in dairy cows. J Dairy Sci.

[CR31] Nemecek T, Jungbluth N, i Canals LM, Schenck R (2016). Environmental impacts of food consumption and nutrition: where are we and what is next?. Int J Life Cycle Assess.

[CR32] Oakes M, Baxter L, Long TC (2014). Evaluating the application of multipollutant exposure metrics in air pollution health studies. Environ Int.

[CR33] OECD-FAO (2018). OECD-FAO agricultural outlook 2018–2027.

[CR34] Oltean-Dumbrava C, Watts G, Miah A (2016). Towards a more sustainable surface transport infrastructure: a case study of applying multi criteria analysis techniques to assess the sustainability of transport noise reducing devices. J Clean Prod.

[CR35] Pimentel BM (2009). Economic costs of water-related health problems in Mexico: deficiencies in potable water services and the costs of treatment of diarrhoeas. Int J Water Resour D.

[CR36] Poore J, Nemecek T (2018). Reducing food’s environmental impacts through producers and consumers. Science.

[CR37] Public Health England (2016). Government dietary recommendations.

[CR38] Ridoutt BG, Hendrie GA, Noakes M (2017). Dietary strategies to reduce environmental impact: a critical review of the evidence base. Adv Nutr.

[CR39] Rodrigues RC (2018). Quiet areas and urban sustainability. Enrgy Proced.

[CR40] Roibás L, Martínez I, Goris A, Barreiro R, Hospido A (2016). An analysis on how switching to a more balanced and naturally improved milk would affect consumer health and the environment. Sci Total Environ.

[CR41] Roy P, Nei D, Orikasa T, Xu Q, Okadome H, Nakamura N, Shiina T (2009). A review of life cycle assessment (LCA) on some food products. J Food Eng.

[CR42] Saarinen M, Fogelholm M, Tahvonen R, Kurppa S (2017). Taking nutrition into account within the life cycle assessment of food products. J Clean Prod.

[CR43] Schau EM, Fet AM (2008). LCA studies of food products as background for environmental product declarations. Int J Life Cycle Assess.

[CR44] Schaubroeck T, Ceuppens S, Luong AD, Benetto E, De Meester S, Lachat C, Uyttendaele M (2018). A pragmatic framework to score and inform about the environmental sustainability and nutritional profile of canteen meals, a case study on a university canteen. J Clean Prod.

[CR45] Scherer L, Tomasik B, Rueda O, Pfister S (2018). Framework for integrating animal welfare into life cycle sustainability assessment. Int J Life Cycle Assess.

[CR46] Schünemann HJ, Sperati F, Barba M, Santesso N, Melegari C, Akl EA, Guyatt G, Muti P (2010). An instrument to assess quality of life in relation to nutrition: item generation, item reduction and initial validation. Health Qual Life Out.

[CR47] Skiba G, Poławska E, Sobol M, Raj S, Weremko D (2015). Omega-6 and omega-3 fatty acids metabolism pathways in the body of pigs fed diets with different sources of fatty acids. Arch Anim Nutr.

[CR48] Sonesson Ulf, Davis Jennifer, Flysjö Anna, Gustavsson Jenny, Witthöft Cornelia (2017). Protein quality as functional unit – A methodological framework for inclusion in life cycle assessment of food. Journal of Cleaner Production.

[CR49] Sonesson U, Davis J, Hallström E, Woodhouse A (2019). Dietary-dependent nutrient quality indexes as a complementary functional unit in LCA: a feasible option?. J Clean Prod.

[CR50] Spadoni M, Voltaggio M, Carcea M, Coni E, Raggi A, Cubadda F (2007). Bioaccessible selenium in Italian agricultural soils: comparison of the biogeochemical approach with a regression model based on geochemical and pedoclimatic variables. Sci Total Environ.

[CR51] Stratton RJ (2007). Malnutrition: another health inequality?: Pennington lecture. Proc Nutr Soc.

[CR52] Stylianou KS, Heller MC, Fulgoni VL, Ernstoff AS, Keoleian GA, Jolliet O (2016). A life cycle assessment framework combining nutritional and environmental health impacts of diet: a case study on milk. Int J Life Cycle Assess.

[CR53] Teixeira R, Himeno A, Gustavus L (2013). Carbon footprint of Breton Pâté production: a case study. Integr Environ Assess Manag.

[CR54] Tessari P, Lante A, Mosca G (2016). Essential amino acids: master regulators of nutrition and environmental footprint?. Sci Rep.

[CR55] Tyszler M, Kramer G, Blonk H (2014). Comparing apples with oranges: on the functional equivalence of food products for comparative LCAs. Int J Life Cycle Assess.

[CR56] Van Kernebeek HRJ, Oosting SJ, Feskens EJM, Gerber PJ, De Boer IJM (2014). The effect of nutritional quality on comparing environmental impacts of human diets. J Clean Prod.

[CR57] Velarde A, Fàbrega E, Blanco-Penedo I, Dalmau A (2015). Animal welfare towards sustainability in pork meat production. Meat Sci.

[CR58] Vieux Florent, Soler Louis-Georges, Touazi Djilali, Darmon Nicole (2013). High nutritional quality is not associated with low greenhouse gas emissions in self-selected diets of French adults. The American Journal of Clinical Nutrition.

[CR59] Warren HE, Scollan ND, Enser M, Hughes SI, Richardson RI, Wood JD (2008). Effects of breed and a concentrate or grass silage diet on beef quality in cattle of 3 ages. I: animal performance, carcass quality and muscle fatty acid composition. Meat Sci.

[CR60] Weidema B, Stylianou K (2019) Nutrition in the life cycle assessment of foods—function or impact? Int J Life Cycle Assess. 10.1007/s11367-019-01658-y

[CR61] Wernet G, Bauer C, Steubing B, Reinhard J, Moreno-Ruiz E, Weidema B (2016). The ecoinvent database version 3 (part I): overview and methodology. Int J Life Cycle Assess.

[CR62] Wood SA, Tirfessa D, Baudron F (2018). Soil organic matter underlies crop nutritional quality and productivity in smallholder agriculture. Agric Ecosyst Environ.

[CR63] Xu Z, Xu W, Peng Z, Yang Q, Zhang Z (2018). Effects of different functional units on carbon footprint values of different carbohydrate-rich foods in China. J Clean Prod.

[CR64] Zuo Y, Zhang F (2011). Soil and crop management strategies to prevent iron deficiency in crops. Plant Soil.

